# The gut microbiome and thermoregulatory response: Relevance of the microbiota in heat‐related illness?

**DOI:** 10.1113/EP093905

**Published:** 2026-05-25

**Authors:** Oliver R. Gibson

**Affiliations:** ^1^ Department of Sport, Health and Exercise Sciences Brunel University of London Uxbridge UK; ^2^ Centre for Physical Activity in Health and Disease (CPAHD) Brunel University of London Uxbridge UK

**Keywords:** exercise, gastrointestinal, heat stress, hyperthermia

1

The impact of heat stress on the human body during physical activity is well established with physiological strain elevated across many organ systems as the body responds to heat storage with the initiation of integrative heat loss pathways (Foster et al., 2026). In circumstances where the combination of the intensity of the activity and the temperature and the humidity of the environment at the skin surface do not allow for sufficient rates of heat dissipation, heat storage leads to increased core body temperature (Foster et al., 2026). In some exercising individuals, excessive heat storage is a factor contributing to exertional heat illness (EHI), a potentially life‐threatening condition. The mechanisms associated with heat‐related illnesses are multifactorial and may demonstrate inter‐individual differences (Gibson et al., 2026); however, the gastrointestinal tract is often implicated (Foster et al., 2026). Hyperthermia, alone or in conjunction with physical activity, can cause gastrointestinal injury, and bacterial translocation into the circulation initiating a systemic inflammatory response and multi‐organ failure. The gastrointestinal tract demonstrates marked differences in functionality between individuals with the gastrointestinal/faecal microbial composition – often referred to as the microbiome – recently suggested as a factor modulating the magnitude of disturbance and inflammatory response during thermoregulatory strain.

To more completely understand this phenomenon, a study in this issue of *Experimental Physiology* utilised a prospective observational cohort design to examine the gastrointestinal microbiota composition and indices of gastrointestinal barrier integrity in individuals who subsequently developed an EHI in comparison to matched non‐EHI control (Gould et al., 2026). A large cohort of participants (*n* = 550, UK military personnel) completed a standardised 6.4‐mile loaded march (∼66 min of exercise in conditions associated with a wet bulb globe temperature (WBGT) of 11 ± 3°C) having provided stool and blood samples in the preceding 3 days. Of the cohort, 79 individuals developed mild or severe EHI and were subsequently grouped and matched to non‐EHI controls, with the authors achieving successful matching across all characteristics except for the ‘by design’ disparity in heat illness symptomatology and core body temperature (controls = 38.9°C; mild EHI = 39.5°C; severe EHI = 39.8°C). Gut microbial composition was analysed via 16S rRNA amplicon sequencing of the stool samples, focusing on diversity and relative abundance with blood‐based biomarkers of gastrointestinal barrier integrity also measured across the cohorts. Interestingly, there were no significant differences in α and β diversity of the gut microbiota, or relative abundance of microbial taxa between EHI cases and controls. A comprehensive assessment of biomarkers associated with alterations in GI barrier integrity, that is, intestinal fatty acid‐binding protein (I‐FABP), claudin 3, zonulin, lipopolysaccharide‐binding protein (LBP) and soluble CD14, showed no significant differences between groups, even in severe EHI cases. The authors concluding that when participant specific confounders are controlled for, gut microbial composition and barrier integrity are not predictive of EHI.

These data generally support complementary work from the wider group, also published recently in *Experimental Physiology*, which also sought to understand whether GI microbiota contribute to EHI aetiology (Gould et al., 2025). In that study, a cross‐sectional design was utilised to compare the faecal microbiome and gastrointestinal barrier integrity, inflammation and thermoregulatory responses between a group of recent EHI patients and matched controls exercising in heat stress generated by a climate chamber (35°C, 44% RH). Following a 60 min standardised ‘heat tolerance assessment’ utilising a treadmill‐based exercise protocol where both intensity as a percentage of ${\dot{V}}_{O_{2}\max}$ and relative metabolic heat production were controlled for, it was reported that heat‐intolerant individuals had higher plasma [sCD14], a marker indicative of microbial translocation, and a lower *Firmicutes:Bacteroidota* ratio compared to heat‐tolerant individuals. Against the backdrop of many comparable microbial and blood biomarker outcomes, it was concluded that the assessment of faecal microbiome composition does not appear warranted to screen for EHI susceptibility despite the ratio of these two most dominant bacterial phyla in the human gut microbiota being different between groups.

Taken together these novel data, acquired using unique experimental designs and gold‐standard methodological approaches, contribute substantially to our knowledge regarding the associations between the host's microbial composition, gastrointestinal resilience to thermal and exertional challenge, and ultimately EHI. Though the primary conclusion at this stage could be that the microbiome does not substantially influence thermoregulatory outcomes and EHI susceptibility, the authors pertinently conclude that more work is required to state this unequivocally. For example, the homogeneous cohort of active, healthy military personnel limits generalizability to populations that may demonstrate greater microbial diversity or more broad physiological profiles. Additionally future research is necessary to explore interactions across differing exercise and thermal modalities and durations, and undertaking an even more comprehensive microbiome analysis including virome and mycobiome components across different regions of the gastrointestinal tract would potentially capture site‐specific differences in the microbiota (Gould et al., 2025, 2026).

Historically, the risk of EHI has been primarily associated with younger individuals exercising under heat stress. However, as climate change intensifies across many regions, the risk now extends to more groups – including older adults, people with chronic health conditions, and those with lower physical fitness – who engage in physical activity during periods of unfamiliar thermal stress (Foster et al., 2026; Gibson & McKenna, 2026). The expanding scope and severity of EHI risk highlights the importance of understanding individual susceptibility to heat‐related illness and developing effective protective strategies, including exploring the potential role of the microbiome as a modulating factor.

## AUTHOR CONTRIBUTIONS

Sole author.

## CONFLICT OF INTEREST

The authors declare they have no conflicts of interest.

## FUNDING INFORMATION

No funding was received for this work.
